# Prevalence of gastrointestinal helminths in *Banaraja* fowls reared in semi-intensive system of management in Mayurbhanj district of Odisha

**DOI:** 10.14202/vetworld.2015.723-726

**Published:** 2015-06-17

**Authors:** Ananta Hembram, M. R. Panda, B. N. Mohanty, C. R. Pradhan, M. Dehuri, A. Sahu, M. Behera

**Affiliations:** 1 Department of Veterinary Parasitology, College of Veterinary Science and Animal Husbandry, Orissa University of Agriculture and Technology, Bhubaneswar, Odisha, India; 2 Department of Livestock Production and Management, College of Veterinary Science and Animal Husbandry, Orissa University of Agriculture and Technology, Bhubaneswar, Odisha, India; 3 Department of Veterinary Pathology, College of Veterinary Science and Animal Husbandry, Orissa University of Agriculture and Technology, Bhubaneswar, Odisha, India

**Keywords:** *Banaraja* fowl, gastrointestinal helminths, prevalence

## Abstract

**Aim:**

Studies on the prevalence of gastrointestinal helminths infection in *Banaraja* fowls of three blocks (Chandua, Shamakhunta and Bangriposi) of Mayurbhanj district in Odisha with respect to semi-intensive system of rearing.

**Materials and Methods:**

A total of 160 *Banaraja* birds (30 males and 130 females) belonging to two age groups (below 1 month age and above 1 month) were examined for the presence of different species of gastrointestinal helminth infection over a period of 1-year. The method of investigation included collection of fecal sample and gastrointestinal tracts, examination of fecal sample of birds, collection of parasites from different part of gastrointestinal tract, counting of parasites, and examination of the collected parasites by standard parasitological techniques followed by morphological identification as far as possible up to the species level.

**Results:**

Overall, 58.75% birds were found infected with various gastrointestinal helminths. Total five species of parasites were detected that included *Ascaridia galli* (25.63%), *Heterakis gallinarum* (33.75%), *Raillietina tetragona* (46.25%), *Raillietina echinobothrida* (11.87%), and *Echinostoma revolutum* (1.87%). Both single (19.15%) as well as mixed (80.85%) infection were observed. Highest incidence of infection was observed during rainy season (68.88%) followed by winter (66.66%) and least in summer season (41.81%). Sex-wise incidence revealed slightly higher occurrence among females (59.23%) than males (56.67%). Age-wise prevalence revealed that chicks were more susceptible (77.77%) than adults (51.30%) to gastrointestinal helminths infection.

**Conclusions:**

Present study revealed that mixed infection with gastrointestinal helminths of different species was more common than infection with single species and season-wise prevalence was higher in rainy season followed by winter and summer. Chicks were found to be more prone to this parasitic infection and a slight higher prevalence among female birds was observed.

## Introduction

Poultry production in India has been constantly growing over the past decades. Now poultry sector is treated as a vital constituent in country’s rural developmental program and a major component of mixed farming. Backyard poultry farming is now emphasized to substantiate the income of small landholders of the rural area.

Indigenous breed of fowls are preferred than exotic breeds due to their natural resistance to various pathogens. *Banaraja* is one such fowl breed which is preferred by farmers for backyard poultry farming. However, certain gastrointestinal helminths are now becoming a potential threat for the fast growing poultry industry. These pathogens result in severe economic losses due to high morbidity and mortality, decreases production, and low meat quality [1]. There is evidence that different production systems bear different risks of parasitic infections for animals and birds [2]. Among the various disease-causing organisms of poultry birds helminths, *viz*. round worms, tapeworms and flukes always persist in farms as well as in non-descript birds and cause a considerable loss to the poultry farmers. Fowl reared under semi-intensive system are as such exposed to infective stage of many helminths. Nowadays *Banaraja* breed of fowl has been adopted by rural people for backyard poultry under semi-intensive system of rearing. No information was available on various helminthic infections of *Banaraja* breed of fowl in this region of the country except for the reports of Mishra *et al.*, [3] and Padhi *et al*. [4] in non-descript breeds of fowls.

Hence, the present study was, undertaken to explore the helminth fauna of *Banaraja* fowls in three blocks (Chandua, Shamakhunta and Bangriposi) of Mayurbhanj district of Odisha where *Banaraja* breed has gained popularity among tribal poultry farmers with a view to institute an effective control measure against the most prevalent and pathogenic parasites in time.

## Materials and Methods

### Ethical approval

The experiment was done in accordance with the guidelines provided by the Institutional Ethical Committee.

### Study area

**Figure-1:**
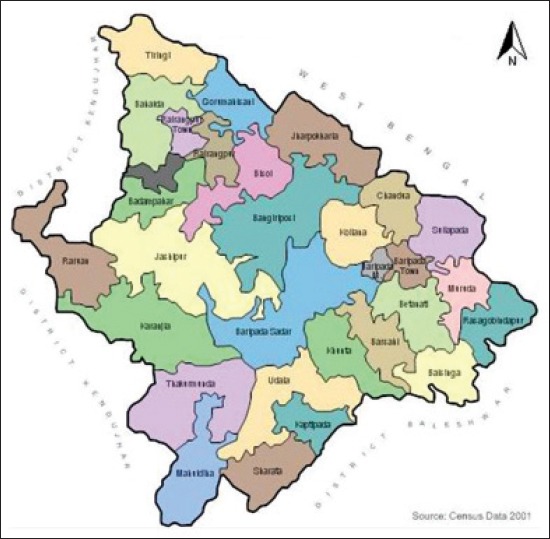
Map of district mayurbhanj, orissa.

The present study was conducted to find out the prevalence of different gastrointestinal helminths infections in *Banaraja* fowls in three blocks (Chandua, Shamakhunta and Bangriposi) of Mayurbhanj district, Odisha (Figure-1).

For a period of 1-year (from July 2012 to June 2013) by examining fecal sample of live birds and gastrointestinal tracts of *Banaraja* birds slaughtered for table purpose.

### Population size

A total of 160 *Banaraja* birds (55 in summer, 45 in rainy, and 60 in winter season) which included 30 males and 130 females belonging to two age groups (below 1 month age and above 1 month age) were examined for the presence of different species of gastrointestinal helminths. *Banaraja* breed of fowls reared by small farmers under backyard farming system were selected for study. Breed, sex, and age of the birds were determined based on respective data obtained from the registers of the supplying sources (Central Poultry Development Organization., Bhubaneswar and Animal Husbandry Department, Government of Odisha) from where the chicks were obtained by the farmers, and also based on external morphology in case of adult birds. Data on sex and age of the birds were recorded individually, which was correlated later while analyzing the observations and results.

### Samples collection

The fecal samples were collected during morning hour while the birds were released from their houses. Fecal samples freshly passed by the birds were collected immediately from the ground avoiding extraneous contamination. Fecal samples from chicks and smaller birds were collected by inducing defecation by gentle massage of vent region of the bird. Samples collected on either way were preserved with 5% formalin in properly labeled screw capped specimen vials and brought to the laboratory for examination. In the laboratory, the samples were examined following standard fecal egg concentration methods such as sedimentation and salt floatation techniques. The worms collected from slaughtered birds were examined following standard parasitologic techniques and identified to genus or species level as per the keys suggested by Soulsby [5].

### Statistical analysis

Statistical analysis was done using Microsoft Excel spreadsheet.

## Results and Discussion

**Figure-2:**
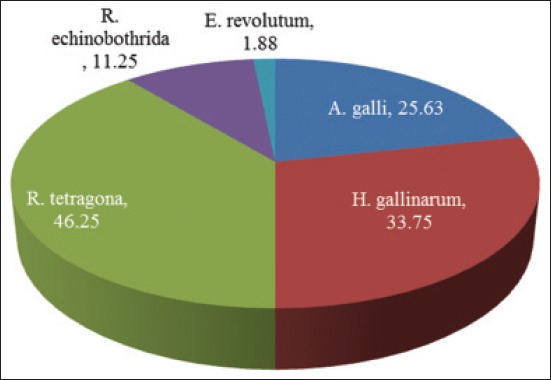
Species wise prevalence of gastrointestinal helminths parasites in *Banaraja* birds.

Present study revealed an overall 58.75% prevalence of gastrointestinal helminthic infection among *Banaraja* breed of fowls reared in the semi-intensive system in Mayurbhanj district of Odisha. The detailed species wise prevalence of gastrointestinal helminths parasites with single and multiple infections has been depicted in Figure-2 and Table-1 respectively. The fowls were infected with two species of nematodes (*Ascardia galli* and *Heterakis gallinarum*), two species of cestodes (*Raillietina tetragona* and *Raillietina echinobothrida*), and one species of trematode (*Echinostoma revolutum*). Katoch *et al*. [6] and Naphade [7] reported an overall 72% and 75.40% prevalence of gastrointestinal helminths among non-descript fowls in subtropical humid zone of Jammu and in Marathawada region of Maharashtra respectively, which were little higher than the present record. Comparatively, still higher rate of incidence of gastrointestinal helminthic infections have been reported by Mishra *et al*. [3], Padhi *et al*. [4], Dehuri [8], and Bal [9] with respective percentage of prevalence of 86%, 100%, 100%, and 94.5% among Deshi fowls in Odisha. Ekpo [10] and Hussen *et al*. [11] while studied the prevalence of gastrointestinal helminths in local breeds in Nigeria and Ethiopia recorded 100% and 89.5% of the incidence, respectively. Presently, lower prevalence rate in *Banaraja* breed of fowls might be due to some kind of natural resistance of these breed of fowls to helminthic infection. Among the infected birds multiple species of parasitic infection was found in 80.85% which has been recorded earlier by Nadakal *et al*. [12] in Deshi birds (Table-1).

**Table-1:** Mixed infection with multiple species of Helminthes in *Banaraja* Fowls.

Species of helminths	Number of infection	Percentage of infection
*Heterakis gallinarum+Raillietina tetragona*	33	20.62
*Ascardia galli+Raillietina tetragona*	18	11.25
*Ascardia galli+Raillietina echinobothridia*	3	1.87
*Ascardia galli+Echinostoma revolutum*	1	0.62
*Ascardia galli+Heterakis gallinarum+Raillietina tetragona*	5	3.12
*Ascardia galli+Heterakis gallinarum+Echinostoma revolutum*	2	1.25
*Ascardia galli+Raillietina tetragona+Raillietina echinobothridia*	2	1.25
*Heterakis gallinarum+Raillietina tetragona+Raillietina echinobothridia*	12	7.50

Of 160 fowls, *R. tetragona* was detected most abundantly (46.25%). Nadakal *et al*. [12] also recorded 67.2% of incidence of *R. tetragona* in Deshi birds. *R. echinobothrida* was also detected causing mixed infection along with other species of gastrointestinal helminths (Table-1). The overall percentage prevalence of *H. gallinarum* and *A. galli* observed during the present investigation were 33.75% and 25.63%, respectively. Comparatively higher prevalence rate of *A. galli* infection in Deshi fowls were reported earlier in Orissa by Padhi *et al*. [4], Mishra *et al*. [3], Dehuri [8] and Bal [9] with percentage of prevalence 47.22%, 81%, 42.65%, and 40%, respectively. Rate of prevalence of *H. gallinarum* and *A. galli* reported previously by Nnadi and George [13] and Rayyan and Al-Hindi [14] were 35.48% and 48.39% in Nigeria, and 68.9% and 75.6% in Palestine, respectively. The low prevalence rate of *A. galli* infection (25.63%) in *Banaraja* breed of fowls observed during the present studies could be attributed to less susceptibility of these birds to *A. galli* infection. *E. revolutum* was only one species of trematode recorded during the present studies. Padhi *et al*. [4] in Orissa had also reported *E. revolutum* from Deshi fowls along with other species of trematodes.

The overall prevalence with gastrointestinal helminths in *Banaraja* birds was highest in rainy season (68.88%) followed by winter (66.66%) and lowest in summer season (41.81%). The low prevalence rate in summer have also been reported by Varghese and Peter [15], Matta and Ahluwalia [16], Padhi *et al*. [4], and Hange *et al*. [1] in Deshi fowl from different places. The decrease in prevalence of parasite in summer might be due to warm climate with low relative humidity prevailing in Mayurbhanj district. These factors are unfavorable for survival of exogenous stage of parasite and accordingly, the bird pick of low infection. The higher prevalence of parasite in the rainy season might be due to increase in number of intermediate hosts and favorable relative humidity.

The rate of prevalence of gastrointestinal helminths among *Banaraja* breed fowls below 1 month of age was recorded higher (77.77%) as compared to (51.30%) in adult birds. This corroborated with the earlier observations made by Padhi *et al*. [4] that the overall prevalence of helminths infection was higher in birds below 6 months age than adult. However, the variation in the rate of prevalence of helminthic infection due to age was not found statistically significant.

The sex wise prevalence of gastrointestinal helminths in *Banaraja* birds showed that 56.67% male and 59.23% female population were infected, but, the variation was not statistically significant. Malhotra and Capoor [17] found 37.74% males and 62.18% female fowls infected with gastrointestinal helminths and Matur *et al*. [18] found higher prevalence rate among females than males of Deshi birds in their study. This might be due to longer reproductive life span of female as compared to male putting them under prolonged reproductive stress. Moreover, females are voracious in their feeding habits especially during egg production than the males which remain largely selective, which could have increased the risk of infection in females [18]. On the contrary, Hirut [19] and Tesfaheywet *et al*. [20] observed that there was no natural affinity of helminths species to either sex of the host.

## Conclusions

The present study revealed that the prevalence of mixed infection was more common than that of single species parasitic infection and season-wise incidence was found to be higher in rainy followed by winter and summer. Chicks were found to be more prone to this parasitic infection and a slight higher incidence among female birds was observed. In spite of minimal health care and improper sanitation practices adopted by the poor backyard poultry farmers rearing *Banaraja* breed of fowls the comparative lower rate of prevalence of gastro-intestinal helminths among *Banaraja* fowls as compared to other non-descript breeds of fowls might be an indicator of possible resistance of this breed to helminthic infection.

## Authors’ Contributions

AH, MRP, BM, and CRP designed the experiment. AH, BM, MD, AS, and MB carried out the experimental work. AH, MRP, BM, CRP, MD, and AS were involved in scientific discussion and analysis of the data. AH, MRP, BM, CRP, MD, AS, and MB drafted and revised the manuscript. All authors read and approved the final manuscript.
